# Editorial: Novel therapeutic strategies for retinal degeneration

**DOI:** 10.3389/fopht.2024.1507844

**Published:** 2024-10-23

**Authors:** Manuel Soliño, Juan J. López-Costa, Alfredo Martínez

**Affiliations:** ^1^ Instituto de Biología Celular y Neurociencia “Prof. E. De Robertis” (IBCN), Universidad de Buenos Aires-Consejo Nacional de Investigaciones Científicas y Técnicas (CONICET), Buenos Aires, Argentina; ^2^ Angiogenesis Group, Center for Biomedical Research of La Rioja, Logroño, Spain

**Keywords:** retinal degeneration, age-related macular degeneration, angiogenesis, therapeutic strategies, gene therapy

Retinal degeneration (RD) is a leading cause of blindness worldwide. The anatomical properties of the eye, as well as its immune privileges, put inherited retinal degeneration (IRD) at the center of the gene therapy revolution. This Research Topic offers, first, a review of classic adeno-associated virus (AAV)-based gene therapies and animal models. It also highlights the second/third generation approaches to gene therapy focusing on gene editing and non-viral vectors.

In Xia et al., the authors wrote a concise update of basic current gene therapies used in clinical trials, animal models, therapeutic windows, vectors and dosage. Among gene therapies, Luxturna for the treatment of Leber amaurosis is the only gene therapy approved so far by the FDA. The choice of a proper animal model is essential to simulate human degenerative diseases. Mice constitute the most widely used model since their retinal structure is similar to humans and they can easily provide knock-out/-down models. The downside of mice is their lack of a macula, the presence of only two types of cones, and the small size of their eyes to perform subretinal manipulations. The rat is also a good model because, although they share some disadvantages with mice, their bigger eyes allow for easier subretinal injections. Rabbits and non-human primates emerge as optimal animal models to assess safety before clinical trials.

Recombinant AAV are the most widely used vectors in gene therapies for the treatment of recessive diseases with loss of function mutations, while RNA-based therapies such as antisense oligo nucleotide (AON) are the elective procedure for dominant diseases with gain of function mutations. When cargo limitations appear, equine anemia virus or nanoparticles may be proper alternatives. Authors also discuss the therapeutic window and dosage testing as important parameters to take into consideration.

**Figure 1:**
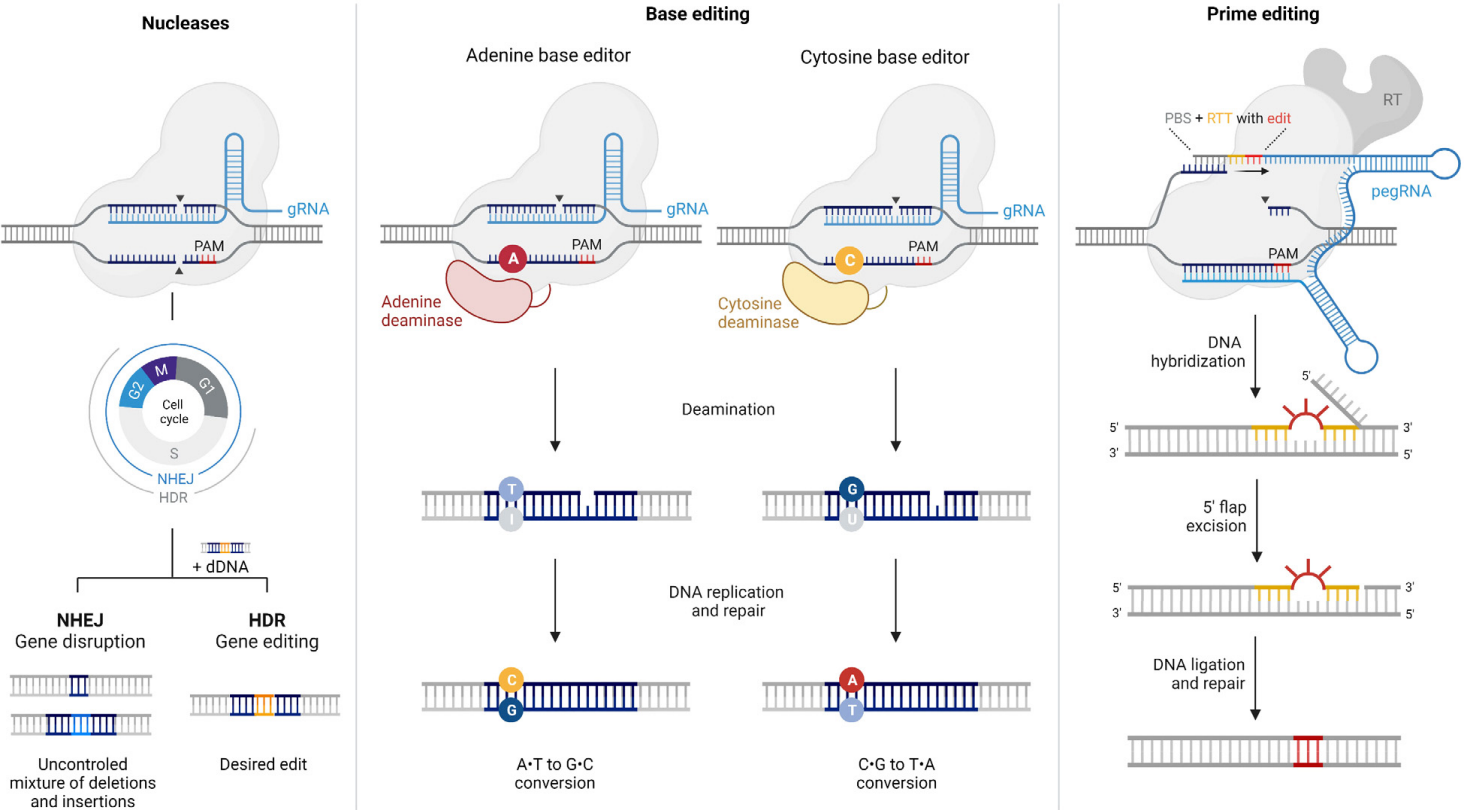
Comparison of CRISPR/Cas9 nucleases, base editors, and prime editors. This figure provides an overview of the components and mechanisms of action of CRISPR/Cas9 nucleases, base editors, and prime editors, highlighting their unique abilities in achieving specific genetic modifications with varying levels of precision. Reproduced, with permission, from the article by Carvalho et al..

In Carvalho et al., the authors summarize new editing techniques, which offer precision and efficiency and bypass immune response when compared to viral vectors. CRISPR-based techniques enable precise modification, removal and/or replacement of target DNA sequences, correct mutations, halt expression of mutant protein and their effects do not decline over time (**Figure 1**). Their therapeutic effect may be extended to dominant diseases. For instance, this approach was used to correct splicing errors in the CEP290 gene, in both mice and non-human primates, and this was the basis for the first Phase I clinical trial approved by the FDA using this technology. Base Editing and Prime Editing are alternative technologies that build upon the more classic CRISP/Cas model with preclinical studies showing highly precise and efficient corrections.

In recent years, nanoparticles appeared as a valuable tool to optimize gene therapies and a wide range of nanocarriers are being explored. A nanoparticle was used for codelivery of CRISPR/Cas9 and RS1 gene in an animal model of X-link juvenile retinoschysis. Recently, viral-like particles (VLPs) emerged as candidates for delivery of genome editing components. Finally, the authors revise physicochemical properties of nanoparticles which facilitate gene delivery and help to overcome eye barriers.

A second group of articles addresses the most common form of retinal degeneration, age-related macular degeneration (AMD), which still puzzles researchers, especially in the case of its most prevalent clinical presentation, geographic atrophy (GA). In this Research Topic, a comprehensive review of the current knowledge of GA, as well as a novel result showing a protective effect for dual antiplatelets in patients, are presented. Wet AMD is characterized by choroidal neovascularization (CNV). The role of Meteorin-like (Metrnl), an endogenous protein down-regulated in patients and able to ameliorate damage in a photocoagulation model of CNV, is also presented.

In Rajanala et al., the authors summarize diagnostic methods, pathophysiology, and novel therapeutics methods for GA, which is the advanced stage of AMD that leads to gradual and permanent vision loss. Among the main risks factors, advanced age, family factors, smoking, and genetic variants are mentioned. Among the pathophysiological pathways, the authors point out the role of the complement cascade in AMD, inflammosome activation, recruitment of microglia and macrophages to the subretinal space, oxidative stress, and genetic factors involved. Up to now, only two treatments that slow down progression of GA have been approved by the FDA (Pegcetacoplan and Izervay) but, unfortunately, no therapy is able to recover the cells that are lost in the retina. On top of the classic treatment options for GA, newer treatments including cell therapies, modulators of visual cycle, and photobiomodulation with low intensity light and their results are also discussed.

In Chantarasorn et al., the authors make a retrospective study about the role of dual antiplatelets treatment in GA secondary to non-vascular AMD. This retrospective cohort study included 96 patients who met inclusion criteria, however only 50 had complete imaging data and two of them had poor adherence to treatment due to peptic ulcers. The control group consisted of 22 patients and 4 of them received 81 mg of aspirin daily. During the studied period, treated patients received 75 mg of Clopidogrel plus 81 mg of aspirin while the control group did not receive this treatment. Damages areas of the retina were determined by red-filtered fundus autofluorescence at 3, 6 and 12 months of the study. Results showed that patients receiving dual antiplatelets were associated with deceleration of GA growth, showing the benefit of this treatment.

In Zhang et al., the authors studied the role of Meteorin-like (Metrnl), a novel cytokine, to attenuate choroidal neovascularization (CNV). The authors found that Metrnl stabilized endothelial function and inhibited angiogenesis in a mouse model of CNV. This molecule interacts with UCHL-1, regulates the NF-kB signaling pathway, and inhibits secretion of inflammatory cytokines by macrophages. Determination of Metrnl in the aqueous humor of wet AMD patients showed an increase of IL-1b and IL-6 and a decreasing trend of Metrnl. The data suggest that Metrnl could constitute a potential drug to prevent the progress of CNV in wet AMD.

In all, this Research Topic covers the state of the art in retinal degeneration and presents some new directions that may change the course of disease in a large group of patients that currently have no therapeutic alternatives.

